# Supplementary material for: PSMD14 Targeting Triggers Paraptosis in Breast Cancer Cells by Inducing Proteasome Inhibition and Ca2+ Imbalance
Source: Int J Mol Sci. 2022 Feb 28;23(5):2648. doi: 10.3390/ijms23052648 (PMC8910635; doi:10.3390/ijms23052648)
Supplement: Supplementary file 1 [file ijms-23-02648-s001.zip › ijms-1592190-supplementary.pdf]

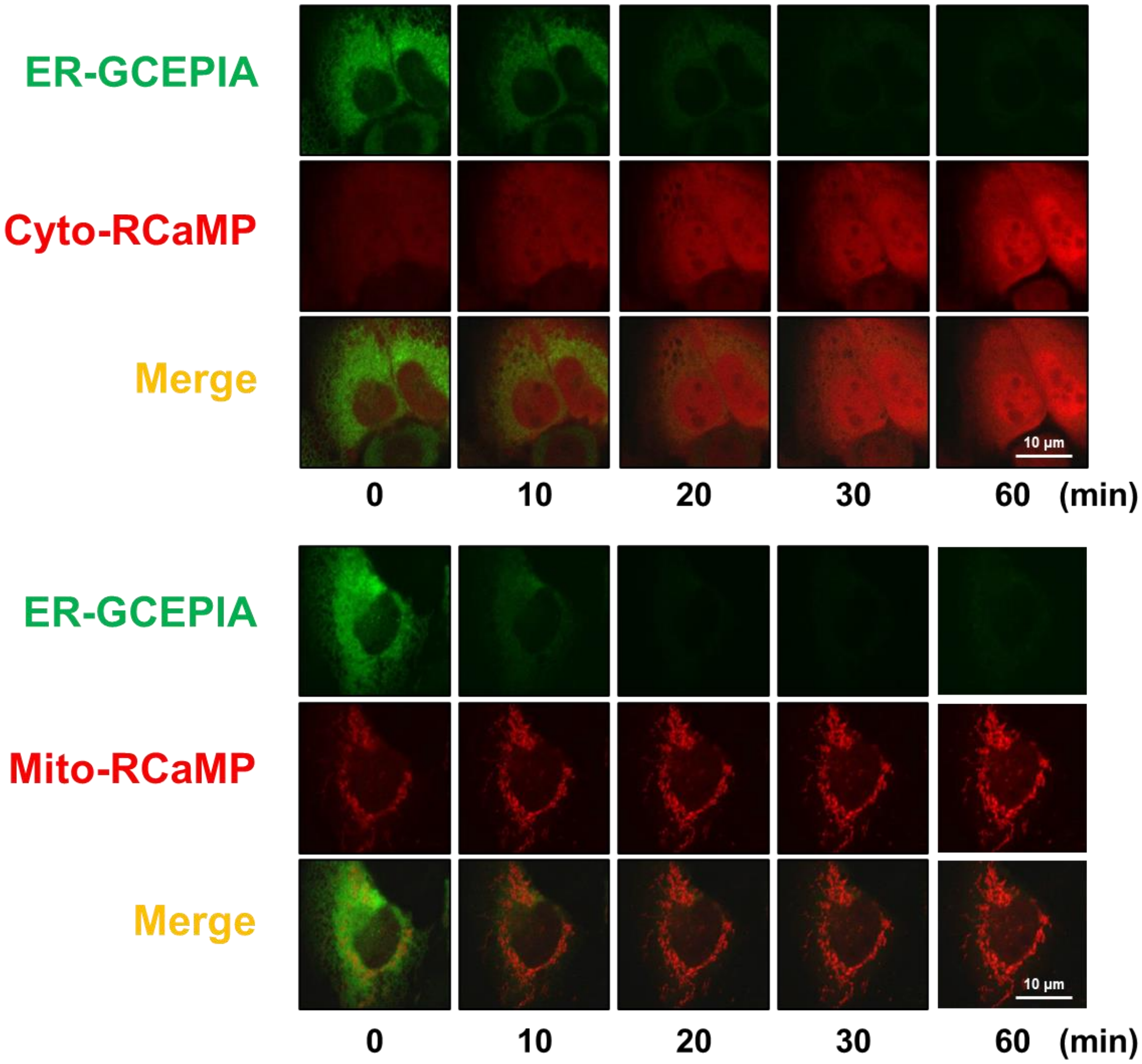

Supplementary Figure S1. Time course subcellular Ca^{2+} dynamics following CZM treatment. MDA-MB 435 S cells co-expressing G-CEPIA1er and cyto-RCaMP1h or cells co-expressing G-CEPIA1er and mito-RCaMP1 h were treated with 5 μM CZM for the indicated time durations. Subcellular Ca^{2+} dynamics were monitore d under the confocal microscope and representative images are displayed. Bars, 10 μm.

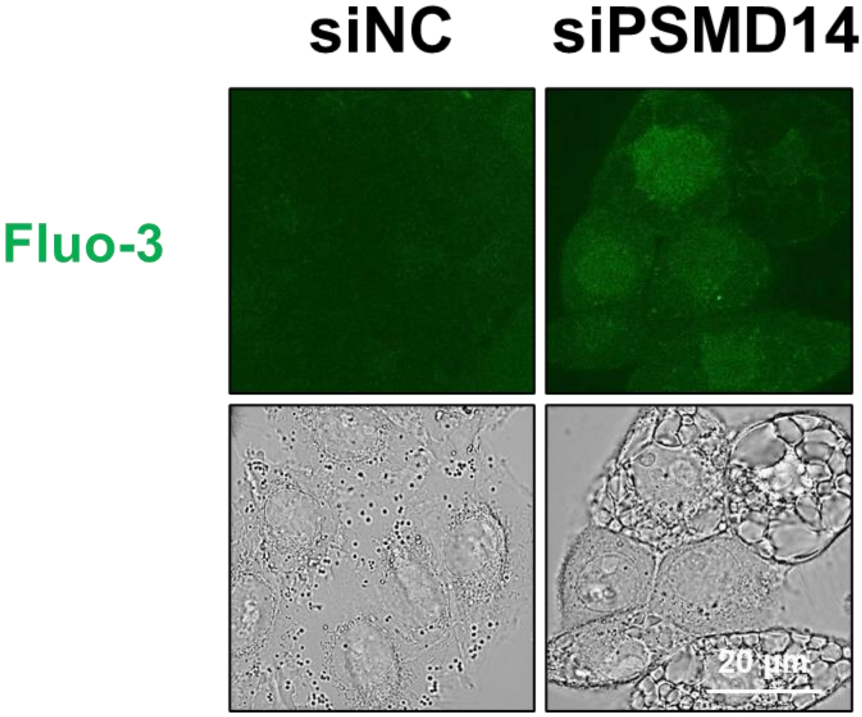

Supplementary Figure S2. Knockdown of PSMD14 increases cytosolic Ca^{2+} levels. MDA-MB 435Scells trans fected with the siRNA against PSMD14 for 36 h and further incubated with the fresh medium were staine d with Fluo-3 and observed under the confocal microscope. Bars, 10 μm.
